# Crisis Concept Re-loaded?—The Recently Described Suicide-Specific Syndromes May Help to Better Understand Suicidal Behavior and Assess Imminent Suicide Risk More Effectively

**DOI:** 10.3389/fpsyt.2021.598923

**Published:** 2021-03-24

**Authors:** Viktor Voros, Tamas Tenyi, Agnes Nagy, Sandor Fekete, Peter Osvath

**Affiliations:** Department of Psychiatry and Psychotherapy, Medical School, University of Pecs, Pecs, Hungary

**Keywords:** suicide-specific syndromes, acute suicidal affective disturbance, suicidal crisis syndrome, suicide risk factors, suicide prediction, suicide prevention

## Abstract

**Background:** Despite of the decreasing suicide rates in many countries, suicide is still a major public health concern worldwide. Traditional suicide risk factors have limited clinical predictive value, as they provide little reliable information on the acute psychological processes leading to suicide.

**Aims:** The aim of this analysis is to describe and compare the recently introduced two suicide-specific syndromes [*Acute Suicidal Affective Disturbance (ASAD)* and *Suicidal Crisis Syndrome (SCS)*] with the classic psychological features of pre-suicidal crisis and also to assess the clinical utility of the new suicide prediction scales in contrast to classical risk factors.

**Method:** Conceptual analysis.

**Results:** Suicide-specific syndromes are not novel in terms of symptomatology or dynamics of symptom onset, but in their use of well-defined diagnostic criteria. In addition to symptomatic classification, they also provide an opportunity to objectively measure the current pre-suicidal emotional and mental state by validated tools.

**Limitations:** Future studies need to be completed to prove the reliability and predictive validity of suicide-specific diagnostic categories and the related suicide risk assessment tools.

**Conclusion:** Clinical use of suicide-specific syndromes is suggested. This transdiagnostic approach not only enables a more accurate and objective assessment of imminent suicide risk, but also facilitates research in neuroscience, which represent a major step forward in managing and complex understanding of suicidal behavior.

## Introduction

Despite of the decrease in the overall global suicide rates in recent decades, suicide is still a common cause of death, especially among younger people. Furthermore, in some countries, for example in the United States an increase of suicide was reported across all age groups (1). Therefore, intense neuroscientific research (including genetic, neurobiological, functional imaging and cognitive research) has begun to more accurately identify the underlying factors for suicidal behavior. For now, a number of characteristics are proved to be associated with suicidal behavior. However, the multicausality of suicidal behavior and the complex development of suicide risk, involving biological, psychological, clinical, social and environmental factors predicts the difficulty of suicide risk assessment (2). Furthermore, risk factors need to be considered at the population and individual levels, and also predisposing and precipitating factors have been distinguished (3).

From a clinical perspective, the hierarchical classification of risk factors (4) differentiates between primary (mental disorders, previous suicide attempt, low serotonin activity, etc.), secondary (early trauma, negative life-events, smoking, etc.) and tertiary (male gender, periods of developmental crises, vulnerable periods, etc.) risk factors. This classification may help to determine the targets and methods of intervention. However, traditional suicide risk factors have only limited clinical predictive value (5), because they provide little reliable information on the acute psychological processes leading to suicide and on imminent suicide risk assessment. According to some researchers, studies on the subjective aspects of suicidal behavior would help to clarify the emotional and psychological background (“psychache”) of suicidal behavior and may lead to a paradigm-shift in suicide risk assessment (1).

Because only weak evidence supports the routine use of currently available assessment tools, new risk assessment models with high negative predictive value should be developed to support clinical decision-making and preserve resources (3). Therefore, the use of structured suicide prediction tools as adjuncts to an individual psychiatric assessment is recommended by the European Psychiatric Association (6). As the lack of precise recognition of acute suicide risk limits the ability to provide adequate care, more research has begun to develop methods for a better risk assessment and complex risk analysis that may provide more accurate predictions (7, 8).

For this reason, many researchers have advocated for the introduction of a suicide-specific diagnostic category in the psychiatric nomenclature and in the diagnostic classification systems for mental disorders (5, 9, 10). The Section III of the Diagnostic and Statistical Manual of Mental Disorder, Fifth Edition (DSM-5) already includes *Suicidal Behavior Disorder* (SBD) among Conditions for Further Study (9, 11) (Table 1). According to the currently proposed criteria, it is defined as an attempted suicide within 24 months. The diagnosis is not applied to suicidal ideation or to preparatory acts, and if the act was initiated during a state of delirium or confusion, and if the act was undertaken solely for a political or religious objective. Other specifiers of suicidal behavior according to the DSM-5 refer to the violence (violent or non-violent) and the lethality (high or low lethality) of the method, and the dynamics (planned or impulsive) of the attempt (11) (Table 1). While these are clinically important features, they alone provide little useful information on the background of suicidal behavior (e.g., mental disorder, crisis situation, etc.) and do not help to identify warning signs or acute risk and to predict future suicidal behavior (12, 13). Because acute suicide risk usually develops rapidly (up to some days or hours) (14), it would be necessary to introduce a category that emphasizes the characteristics of this life-threatening pre-suicidal state of mind requiring urgent intervention (15, 16). Furthermore, in the recent, 11th Revision of the International Classification of Diseases (ICD-11), suicidal behavior is also listed outside the chapter on mental, behavioral, or neurodevelopmental disorders (10). In ICD-11, the different forms of self-injuries, self-harming and suicidal behavior are listed in different chapters, such as External Causes of Morbidity or Mortality, or Symptoms, Signs, and Clinical Findings not Elsewhere Classified (10).

**Table 1:** The diagnostic criteria for suicidal behavior disorder (SBD), according to the DSM-5 (Section III, Conditions for Further Study) (11).

A. Within the last 24 months, the individual has made a suicide attempt. Note: A suicide attempt is a self-initiated sequence of behaviors by an individual who, at the time of initiation, expected that the set of actions would lead to his or her own death. The “time of initiation” is the time when a behavior took place that involved applying the method.) B. The act does not meet criteria for non-suicidal self-injury — that is, it does not involve self-injury directed to the surface of the body undertaken to induce relief from a negative feeling/cognitive state or to achieve a positive mood state. C. The diagnosis is not applied to suicidal ideation or to preparatory acts. D. The act was not initiated during a state of delirium or confusion. E. The act was not undertaken solely for a political or religious objective. *Specify* if: Current: Not more than 12 months since the last attempt. In early remission: 12–24 months since the last attempt.

*Other specifiers of suicidal behavior are the violence of the method (violent or non-violent), the lethality (high or low) and the dynamics (planned or impulsive) of the attempt*.

**Figure 1:**
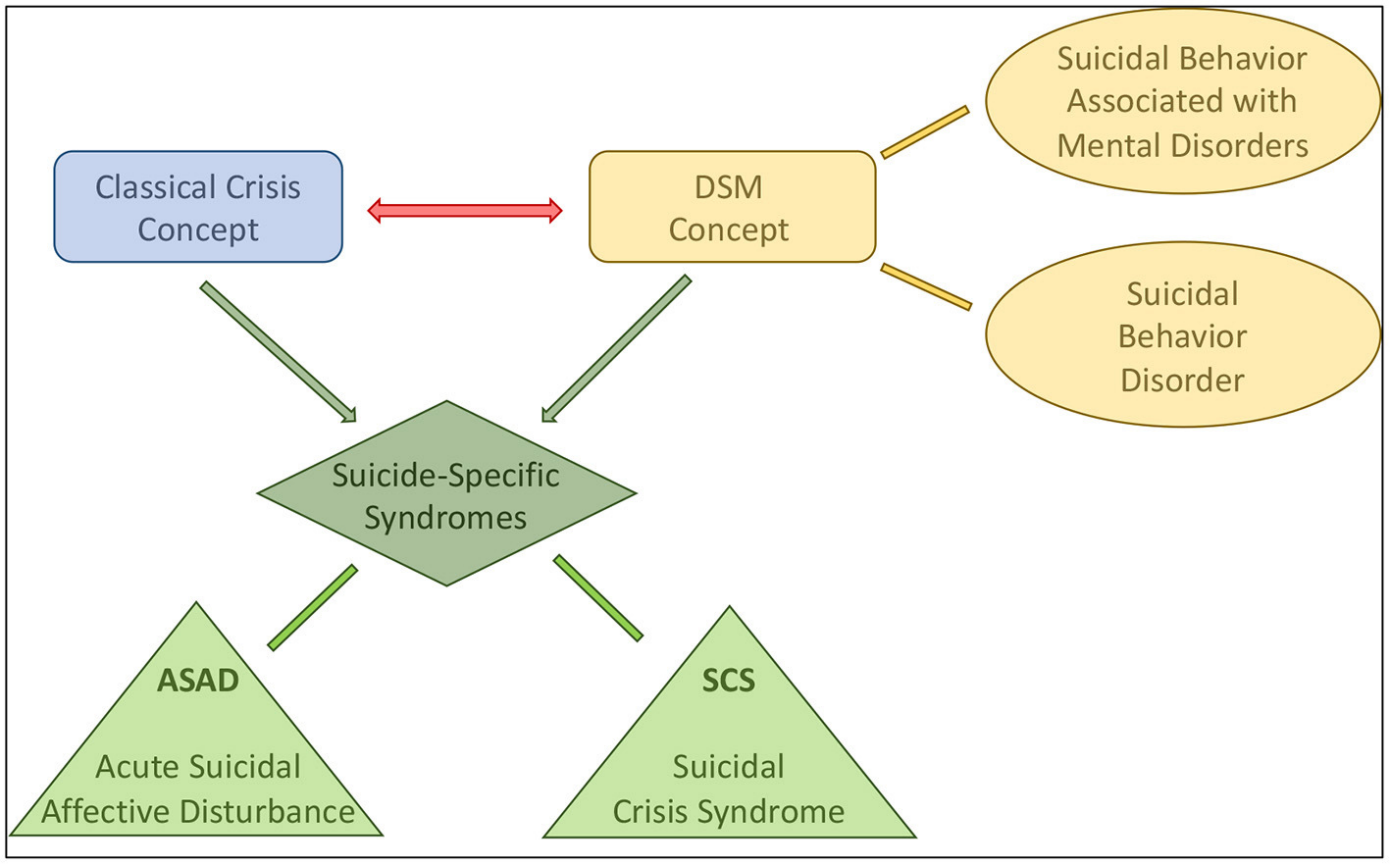
Concept and development of the newly described suicide-specific syndromes [acute suicidal affective disturbance (ASAD) and suicidal crisis syndrome (SCS)]. The DSM-5 involves suicidal behavior disorder in its Appendix. Suicidal behavior may also be associated with different mental disorders, such as major depressive disorder, borderline personality disorder, etc. In contrast, the classical crisis theory, as a transdiagnostic concept, focuses on the emotional and psychological aspects of suicidal behavior, regardless of their potential association with mental disorders. Suicide-specific syndromes integrate these two major concepts with establishing well-defined diagnostic criteria, but also consider the acute psychological and psychopathological changes during the pre-suicidal period. Two different forms of suicide-specific syndromes were described recently, the ASAD and the SCS.

However, in recent years, two complex, specific syndromes have been described that may assist in a more accurate assessment of pre-suicidal psychopathology and thus in the prediction of suicidal behavior. Researchers recommended the introduction and—after a predictive validation process—the clinical use of two suicide-specific syndromes, the *Acute Suicidal Affective Disturbance (ASAD)* (13, 17) and *Suicidal Crisis Syndrome (SCS)* (5, 18) (Table 2). These newly described syndromes deserve attention because the well-known, classical theory of suicidal crisis by Caplan and the presuicidal syndrome, described by Ringel (Ringel's Triad), as well as other related phenomena, such as “psychic pain,” “cry-for-help,” or “cry-of-pain” have been already known for decades (20) (Table 3). This classical description of suicidal crisis forms basis of understanding the subjective experiences of patients with suicidal behavior, that need to take into account when planning effective risk assessment and suicide prevention methods (1). The concept of suicide-specific syndromes combines this traditional crisis theory with the structured diagnostic concept of the recently used major diagnostic and classification systems, such as the DSM-5 (Figure 1).

**Table 2:** Proposed brief diagnostic criteria for suicide-specific syndromes by Joiner (acute suicidal affective disturbance (ASAD) and Galynker (suicide crisis syndrome (SCS) (19).

**ASAD**	**SCS**
A. A drastic increase in suicidal intent over the course of hours or days, as opposed to weeks or months B. One (or both) of the following: marked social alienation (e.g., social withdrawal, disgust with others, perceptions that one is a liability on others) and/or self-alienation (e.g., self-hatred, perceptions that one's psychological pain is a burden) C. Perceptions that one's suicidality, social alienation, and self-alienation are hopelessly unchangeable D. Two (or more) manifestations of overarousal (i.e., agitation, irritability, insomnia, nightmares)	A. Persistent or recurring feeling of entrapment and urgency to escape or avoid a perceived inescapable and unavoidable life situation. Although death may appear as the only escape, explicit suicidal ideation need not be (though may be) present B. Affective, behavioral, and cognitive changes associated with the experience of entrapment, including at least 1 item from a to d: a. Affective disturbance b. Loss of cognitive control c. Disturbance in arousal d. Social withdrawal

**Table 3:** Comparison of the newly described suicide-specific syndromes [acute suicidal affective disturbance (ASAD) and suicidal crisis syndrome (SCS)] with suicidal behavior disorder (SBD) included in DSM-5 and with the classical suicidal crisis concept.

	**Suicide-specific syndromes**		**DSM-5 category**	**Classical crisis concept**
	**ASAD**	**SCS**	**SBD**	**PSS**
Term	Acute Suicidal Affective Disturbance	Suicidal Crisis Syndrome	Suicidal Behavior Disorder	Pre-Suicidal Syndrome (Suicidal Crisis)
References	Tucker et al. (17)	Galynker (18)	DSM-5 (11)	Ringel (21) and Caplan (22)
Key-symptom	- Drastic increase in suicidal intent over the course of hours or days	- Persistent or recurring feeling of entrapment	- Suicide attempt within the last 24 months	- Ringel-triad: - Constriction - Inhibited aggression turned toward the self - Suicidal fantasies
Other major characteristics	- Social and self-alienation - Hopelessness - Hyperarousal	- Affective and cognitive dysregulation with behavioral symptoms: - Affective disturbance - Loss of cognitive control - Disturbance in arousal - Social withdrawal	- Not applied: - To suicidal ideation or preparatory acts - If initiated during delirium or confusion - If undertaken solely for political or religious objective	- Caplan's crisis concept: - Perceive an event as being threatening - Unable to modify or lessen the impact - Increased fear, tension, confusion - High level of discomfort - State of disequilibrium - Other symptoms: - Regression - Autonomic symptoms - Insomnia - Psychomotor symptoms - Behavior changes
Course, dynamics	- Rapid (up to hours or days) - Spike-like (brief and intense) - Time-limited	- Persistent or recurring	- Planned (chronic) or impulsive (acute) - 24 months	- Fluctuating - Vortical

We present the most important features of these newly described suicide-specific syndromes, the experience with their clinical application and the major research findings. Then these syndromes are compared with each other and with the classical psychological features of pre-suicidal crisis to find out whether these are new interpretations of suicidal behavior or those are merely the well-known classical symptoms with new terminology (“old wine in new bottles”).

## Acute Suicidal Affective Disturbance and Suicidal Crisis Syndrome

*Acute Suicidal Affective Disturbance* (ASAD) is based on empirical pre-suicidal clinical features and theory-driven predictors (13, 17, 19) (Table 2). This concept emphasizes that symptoms develop very quickly. Its main components are: drastic increase in suicidal intents over the course of hours or days; marked social alienation (e.g., social withdrawal, disgust with others, perceived burdensomeness) and/or self-alienation (self-hatred, perceptions that one's psychological pain is a burden); the above mentioned are hopelessly unchangeable; and over-arousal (agitation, irritability, insomnia, nightmares) (12). A 28-item clinical scale (ASADI-L) was also developed to assess lifetime ASAD symptoms (17). The validity of these symptoms and their distinction from other psychiatric disorders have been demonstrated in numerous studies, including healthy population and people with different mental disorders (16, 23, 24). The construct validity of ASAD as a unified entity was first demonstrated by Tucker et al. (17) in a sample of undergraduate students. A confirmatory factor analysis with 1,442 psychiatric in-patients replicated previous results and confirmed the factor structure of ASAD in a large sample (16). Later, the unidimensional nature, and also the convergent and discriminant validity of ASAD were demonstrated in a large sample of out-patients with mental disorders (23). According to these studies, ASAD seems to be a unified construct that may predict suicidal behavior and can be clearly differentiated from other mental disorders, including major depressive disorder. Moreover, ASAD not only significantly predicted a past suicide attempt (16), but also differentiated multiple attempters, single attempters and non-attempters as well as attempters, ideators, and non-suicidal patients (23).

*Suicidal Crisis Syndrome* (SCS) (18) is a presuicidal mental state with affective and cognitive dysregulation and behavioral changes in response to a real or perceived threat (19, 25) (Table 2). The symptoms occur unexpectedly when the individual is unable to cope with a situation, which is unacceptable, intolerable and unescapable (such as loss of a job or love relationships, etc.). The key symptoms of SCS are the persistent or recurring feeling of entrapment and urgency to escape from a perceived unavoidable life situation (12, 25). Thus, death may appear as the only escape, however explicit suicidal ideation need not be (though may be) present. Other diagnostic criteria include affective symptoms (affective disturbances as manifested by emotional pain, rapid spikes of negative emotions, extreme anxiety, acute anhedonia); cognitive impairments (loss of cognitive control as manifested by rumination, cognitive rigidity, ruminative flood/cognitive overload, repeated unsuccessful attempts to suppress negative or disturbing thoughts); and behavioral changes [as manifested by disturbance in arousal (agitation, hypervigilance, irritability, insomnia) and/or social withdrawal (reduction in social activity, evasive communication)] associated with the experience of entrapment (12). These symptoms develop rapidly and may last minutes to days, and then may persist or recur. As the condition progresses, symptoms may either increase in their intensity or fluctuate rapidly, or the two types may mix. Social isolation is often associated with the syndrome, which further increases the risk of suicide. The coherence and predictive validity of these symptoms have also been confirmed by numerous studies (25). Galynker et al. (5) also developed an assessment tool, the Suicide Crisis Inventory (SCI) to measure SCS. The SCI had significantly more pronounced predictive value for suicidal behavior in high-risk psychiatric patients after discharge from the hospital than traditional, classical suicide risk factors (5, 18). In this respect, the subscale of “feeling of entrapment” had the most powerful predictive value (5). The clinical utility of SCI is strongly supported by the evidence, that SCI was predictive of future suicidal behavior after discharge, thus it may help clinicians in their decision-making regarding the termination of the hospitalization (5). In addition to this clinical benefit, the use of SCI may have a positive impact on the related potential legal and economic conditions.

## Comparison of the Suicide-Specific Syndromes

The two suicide-specific syndromes show a number of similarities, particularly in terms of their concept of the dynamics of suicidal behavior, as the symptoms are acute and rapidly worsening and precede suicidal behavior in both cases (Table 3). They are also characterized by hyperarousal, hopelessness and social withdrawal (12), all contributing to an increase in acute suicide risk (25). ASAD is characterized by rapid and extreme increase of conscious suicidal intents, accompanied by social withdrawal, hopelessness, and hyperarousal, while the key symptom of SCS is the feeling of entrapment, even without direct suicidal intents (19, 25, 26). In SCS, the loss of cognitive control results in the impairment of the executive functions, leading to a further deterioration of the problem-solving and coping capacity. This is the background to the dynamically occurring mental process in which suicide may seem to be the only possible solution. While ASAD is characterized by a rapid escalation of the basic symptoms, in SCS the progressive or fluctuating course of the associated affective, cognitive and behavioral symptoms and hyperarousal are highlighted. Furthermore, social withdrawal and isolation are key symptoms in ASAD, whereas in SCS those are only considered as accessory phenomena. Similarly, hopelessness, which creates an intense desire to escape the situation at all costs is in the focus of SCS, whereas in ASAD, in this respect is only incidental. Hyperarousal plays an important role in both syndromes, but in ASAD nightmares are more prominent, whereas in SCS, hypervigilance is emphasized (Table 3).

Based on the above, it can be concluded that there are many overlaps between the two new suicide-specific syndromes and also with the symptoms of the classical suicidal crisis. Cognitive (e.g., feeling of being unable to cope, cognitive distortions, futility, hopelessness); affective (e.g., depression, anxiety, emotional instability); behavioral (e.g., narrowing of the repertoire of actions, inadequate problem-solving capacity); vegetative (e.g., insomnia, somatic complaints associated with anxiety); psychomotor (e.g., regression or agitation) symptoms and social characteristics (reduced social relationships) show that most of the features of ASAD and SCS are also found in the classical description of the pre-suicidal syndrome (Table 3).

## Discussion

As with the classical crisis paradigm (20, 27), new suicide-specific syndromes have the great advantage that the detailed assessment of the characteristics and dynamics of symptom presentation may provide a basis for a more accurate risk assessment and intervention. Accurate and routine implementation of a scientifically proven risk assessment is the best way to effectively evaluate and manage suicide risk. Applying and adequately documenting these methods as the “gold standard” can also prevent possible negative legal consequences (12).

Before suicide-specific syndromes are considered as clinically useful and validated diagnostic entities included in the diagnostic systems of mental disorders, future studies need to be completed to prove their reliability and predictive validity. It is also an important goal for future research to find out whether these syndromes are independent from other mental disorders and from each other. As these two clinical entities capture different aspects of the pre-suicidal process, further clinical use and research may lead to the description of an integrated syndrome, whose statistically validated criteria combine the symptoms of the two separate suicide-specific syndromes (12). Further research is also needed to identify the symptoms with the best prognostic value and to describe the dynamics of the symptoms more accurately (26). To reach this goal, it is essential to accurately describe the precipitating factors, the clinical features (e.g., suicide-specific rumination), the underlying neurobiological and neuropsychological factors (increased attention to negative emotional stimuli, impaired problem-solving and decision-making, decreased verbal fluency, etc.) in suicidal behavior. Numerous studies have already demonstrated the role of the prefrontal cortex (especially dorsal regions), the anterior cingulate cortex, and the amygdala in the development of suicidal behavior. Neuroscientific research has increasingly outlined the components of neurobiological dysregulation underlying ASAD (28). Based on these findings, certain elements of these syndromes may represent endophenotypic domains that provide a more accurate understanding of the neurobiological background of the complex pre-suicidal emotional state. Furthermore, the application of the Research Domain Criteria (RDoC) approach in suicidology can also help to integrate research results and clarify their clinical relevance (29).

Thus, the description of suicide-specific syndromes may represent a paradigm-shift in the psychological-psychiatric interpretation of suicidal behavior (Figure 1). Previously, in case of many non-fatal or fatal suicide attempts, no mental disorder was identified in the background of the suicidal act, but those were conceptualized as psychological-emotional crisis, based on the traditional crisis theory. However, suicide-specific syndromes condense the complex psychological and psychopathological state and the behavior associated with the suicidal act into a diagnostic category, defining it as a mental disorder. Thus, a significant proportion of suicidal behaviors may still be interpreted as symptoms associated with other major mental disorders (e.g., depression, bipolar disorder, borderline personality disorder, schizophrenia, etc.), but another significant proportion now may be interpreted not only in the framework of the traditional crisis concept, but as the leading symptoms of suicide-specific syndromes. At the same time, suicide-specific syndromes, whether ASAD or SCS, form the basis of a clinically useful transdiagnostic algorithm for detecting imminent suicidal threat. Thus, suicide-specific syndromes may help in a more accurate assessment of suicidal risk, in a more effective prediction of suicidal behavior, and may provide a basis for more effective interventions (12, 13, 19, 26, 30, 31).

As suicidal behavior is a multicausal phenomenon, an integrated approach and modern tools need to be applied for complex risk assessment on a population level (2). These novel methods may include genetic testing, digital phenotyping, data-driven machine learning approach, machine-learning of electric health records, or Computerized Adaptive Testing (CAT) (2, 3, 15, 32). Although, preliminary results with these new strategies on suicide risk assessment and prediction are promising, further testing and randomized controlled trials are needed to assess their effect and clinical validity (2). The suicide-specific syndrome concept may be successfully integrated in this complex approach. Furthermore, the validated factors and clinical tools developed in the framework of the suicide-specific syndrome research may be used not only in the clinical practice, but also in future clinical trials.

In conclusion, compared to the traditional crisis theory, suicide-specific syndromes are not novel in terms of symptomatology or dynamics of symptom onset, but in their use of more well-defined diagnostic criteria. In addition, past suicide attempts and other classical suicide risk factors provide only marginal improvement of diagnostic accuracy and minimal incremental prediction of future suicide attempts (19, 33), but suicide-specific syndromes may also provide an opportunity to objectively measure the current pre-suicidal emotional and mental state by validated clinical tools. Furthermore, as researchers suggest, suicidal behavior (as a suicide-specific syndrome) should be officially recognized in psychiatric nosology, as an independent, codable entity and as a distinct diagnostic category in the major diagnostic and classification systems, such as DSM or ICD (10). This transdiagnostic approach not only enables a more accurate assessment of suicide risk and prediction of suicide, but also facilitates clinical and neuroscientific (neurobiological and neurocognitive studies) research and also the psychological and narrative interpretations of suicidal behavior, which represent a major step forward in managing and complex understanding of suicidal behavior.

## Author Contributions

VV and PO designed and wrote the manuscript. TT raised the concept and reviewed the manuscript. AN and SF helped with the concept and completing the paper and reviewed the manuscript. All authors contributed to the article and approved the submitted version.

## Conflict of Interest

The authors declare that the research was conducted in the absence of any commercial or financial relationships that could be construed as a potential conflict of interest.
